# Assessment of Markers of Antimalarial Drug Resistance in *Plasmodium falciparum* Isolates from Pregnant Women in Lagos, Nigeria

**DOI:** 10.1371/journal.pone.0146908

**Published:** 2016-01-25

**Authors:** Chimere Obiora Agomo, Wellington Aghoghovwia Oyibo, Colin Sutherland, Rachael Hallet, Mary Oguike

**Affiliations:** 1 Malaria Research Laboratory, Nigerian Institute of Medical Research, Lagos, Nigeria; 2 *ANDI Centre of Excellence for Malaria Diagnosis/*WHO/TDR/FIND Malaria Specimen Bank Site, College of Medicine, University of Lagos, Idi-Araba, Lagos, Nigeria; 3 Department of Immunology, London School of Tropical Medicine and Hygiene, London, United Kingdom; Swiss Tropical & Public Health Institute, SWITZERLAND

## Abstract

**Background:**

The use of antimalarial drugs for prevention and treatment is a major strategy in the prevention of malaria in pregnancy. Although sulphadoxine-pyrimethamine (SP) is currently recommended for intermittent preventive treatment of malaria during pregnancy in Nigeria, previously used drugs for prophylaxis such as chloroquine (CQ) and pyrimethamine are accessible as they are purchased over the counter. This study describes the markers of absence or presence of resistance to quinoline (*Pfcrt* and *Pfmdr 1)* and type 1 antifolate antimalarial medicines (*Pfdhfr*).

**Methods:**

*Plasmodium falciparum-*positive dried blood spots from pregnant women attending antenatal clinics for the first time during current pregnancy were investigated for the presence of mutations at codons 72–76 of *Plasmodium falciparum* chloroquine resistance transporter *(Pfcrt)* gene by real time polymerase chain reaction (PCR) using haplotype-specific probes. PCR followed by sequence analysis was used to identify mutations at codons 86, 184, 1034, 1042 and 1246 of *P*. *falciparum* multi-drug resistance-1 (*Pfmdr1)* gene; and codons 16, 50, 51, 59, 108, 140 and 164 of *Pfdhfr* gene.

**Results:**

Two haplotypes of Pfcrt (n = 54) were observed: CVMNK 13(24.2%) and CVIET 41 (75.9%) of the samples. The SVMNT haplotype was absent in this population. The Pfmdr1 (n = 28) haplotypes were NYSND 15(53.6%), YYSND 5(17.9%), NFSND 6(21.4%) and YFSND 2(7.1%). The Pfdhfr (n = 15) were ACNCSVI 4(26.7%), and ACICNSVI 1(6.7%) and ACIRNVI 10 (66.7%). The rate of occurrence of Pfcrt 76T, Pfdhfr108N, Pfmdr186Yand184F were 75.9%, 73.3%, 25% and 28.1% respectively. The Pfmdr1 86Y was associated with low parasitaemia (median = 71 parasites/μl, P = 0.024) while Pfcrt 76T was associated with young maternal age (mean 24.1 ± 4.5 years; P = 0.006). The median parasitaemia were similar (P>0.05) in wild and mutant strains of Pfcrt 76, Pfmdr1 184 and Pfdhfr 108. There was no association between gravidity or gestational age of the women and presence of mutations in the Pfcrt, Pfmdr1 or Pfdhfr genes (P>0.05).

**Conclusion:**

Markers of resistance to chloroquine and pyrimethamine were high, whereas cycloguanil-resistance marker was not present in the studied population. The low level of mutations in the *Pfmdr1*gene indicates likely efficacy of amodiaquine against malaria in pregnancy.

## Introduction

The main strategies for the prevention of malaria during pregnancy in Nigeria are by chemoprophylaxis using sulphadoxine-pyrimethamine (SP) and use of long-lasting insecticide treated nets. In spite of the recommendation of two treatment doses of SP for intermittent preventive treatment of malaria during pregnancy[1], other antimalarial medicines such as CQ, pyrimethamine and proguanil are still used in many antenatal clinics. A wide range of un-recommended antimalarial medicines are available and sold over the counter in medicine stores in Nigeria and are accessible to multigravid pregnant women who continued with medications used during their previous pregnancies. Healthcare providers also prescribed un-recommended antimalarials due to poor knowledge on best practices, low confidence on the safety of SP, and challenges in the institution of on-the-job supervision by stakeholders, such as the Ministry of Health, that ensures compliance with recommend malaria in pregnancy guidelines.

Surveillance of antimalarial drug resistance markers in *Plasmodium falciparum* populations is an important tool in attempts aimed at predicting the level of resistance to antimalarial drugs. Mutations in specific genes of *Plasmodium falciparum* that confer resistance to antimalarial drugs are selected by sustained drug pressure [2]. The emergence and spread of drug resistance depends, in part, on the number of mutations required to encode resistance and their effects on parasite fitness [3]. Specific multiple point mutations in a gene make up a resistance marker for an antimalarial drug.

The level of susceptibility of *P*. *falciparum* strains to quinoline antimalarial drugs such as CQ, amodiaquine, lumefantrine and mefloquine has been attributed to mutations in *Pfcrt* and *Pfmdr1* genes [4,5]. Resistance to type 1 antifolates (pyrimethamine, chlorproguanil, trimethoprim) has been associated with amino acid substitutions in the *Pfdhfr* gene while resistance to type II antifolates (sulfonamides: sulfadoxine and dapsone) are associated with mutations in *P*. *falciparum* dihydropteroate synthase (*Pfdhps*) gene [6,7,8].

The protective efficacy of SP during pregnancy in Lagos, Nigeria has been reported in a longitudinal study [9], but for ethical reasons, the efficacy of non-recommended antimalarial drugs in pregnancy can only be monitored by the elucidation of markers suggestive of resistance to these medicines. We describe here, single nucleotide polymorphisms (SNP) in *Pfcrt*, *Pfmdr1* and *Pfdhfr* genes of *P*. *falciparum* isolates from asymptomatic pregnant women in Lagos, Nigeria, in an attempt to make some inference on the efficacy of SP and non-recommended antimalarials based on the presence or absence of resistance markers.

## Methods

### Study samples and area

Mutations in *Pfcrt*, *Pfmdr1*and *Pfdhfr* genes were investigated in 54 dried blood spots from pregnant women positive for falciparum malaria by microscopy but asymptomatic for malaria. All the women were residents of Lagos metropolis who were recruited on their first antenatal visit to 2 hospitals in central and east zones of Lagos state. The study population was part of the base-line study population that was followed up in a parasitological assessment of pregnant women receiving SP in Lagos, Nigeria [9].

The study was conducted in accordance with principles enshrined in the 1964 Declaration of Helsinki as amended as well as provisions encapsulated by the Nigeria Health Research Ethics Code for research involving human participants. Essentially, the aim and procedures for the research was explained to them with provision of willingness to participate or withdraw at any point of the study without affecting the standard care they should receive in the health facilities. Thus, they gave written informed consent to participate using the template approved by the Ethics Committee before the commencement of the study. Enrolment questionnaires and consent documents were reviewed alongside with the protocol submitted to the Ethics Committee. Following National Guideline on the Prevention of Malaria in Pregnancy, pregnant women at booking at the second trimester after first movement of the foetus has been noticed were given standard dose of SP. All consent documents were storedseparately from other study tools in an assess-controlled cabinet. The study protocol was approved by Ethics Committees of the Nigerian Institute of Medical Research, Lagos and College of Medicine of the University of Lagos, Lagos, Nigeria.

Lagos state is situated in the Southwest region of Nigeria bordered by the Atlantic Ocean. Transmission of malaria is moderate and stable in Lagos but peaks during the wet season corresponding to increase in the population of mosquitoes. Lagos has been described as mesoendemic during the dry season [10].

#### Malaria parasite DNA extraction from dried blood spots

A section of blood spot was cut directly into a 1ml 96 well plate using a sterile Harris Uni-Core hole punch (~3mm diameter). 1mL 0.5% saponin in 1x phosphate buffered saline (PBS) was added into the well and incubated overnight at 37°C to release haemoglobin, leaving parasite DNA on the paper. After a brief centrifugation (4000 rpm for 5 mins) in a plate centrifuge (Eppendorf Centrifuge 5810R) the saponin solution and debris are removed using a vacuum pump. Then 1mL 1x PBS was used to wash the paper twice. 150μL 6% chelex suspension was added to the 96 well plate and plate covered with foil and heat sealed. The plate was incubated in a water bath at 100°C for 25 mins to release parasite DNA from paper. The plate was centrifuged in a plate centrifuge for 2 mins to spin down chelex. The DNA supernatant (~100μL) was transferred to a new plate and stored at -20°C. [11].

#### Amplification of the *Pfcrt* gene

A multiplex real-time polymerase chain reaction (RT-PCR) assay using the Rotorgene 3000 platform (Corbett Research, Australia) was used to genotype the *Pfcrt* gene as described by Sutherland *et al*. [12]. Briefly, *Pfcrt* DNA was amplified from each sample using primers *Pfcrt* F (TGG TAA ATG TGC TCA TGT GTT T) and *Pfcrt* R (AGT TTC GGA TGT TAC AAA ACT ATA GT) in the presence of each of the three double-labelled probes, TaqMan probes (MWG, Germany), representing the wild-type and the two most common resistance-associated haplotypes at codons 72–76 of *Pfcrt*. The probes were CVMNK (50FAM-TGT GTA ATG AAT AAA ATT TTT GCT AA-BHQ1), CVIET (50JOE-TGT GTA ATT GAA ACA ATT TTT GCT AA-BHQ1), and SVMNT (50ROX-AGT GTA ATG AAT ACA ATT TTT GCT AA-BHQ2). The reaction conditions were 95°C for 6 minutes; (95°C for 15 seconds, 55°C for 1 minute) x45 cycles. The different fluorescent molecules enabled the detection of the haplotypes present in each sample. Samples were considered positive for a particular genotype if a CT value of 35 cycles or fewer was obtained in at least two independent PCR experiments. The sequence-specific positive control DNA samples, 3D7 (CVMNK), Dd2 (CVIET), and 7G8 (SVMNT) obtained from the Malaria Research and Reference Reagent Resource (MR4, Manassas, Vermont, USA). Nuclease-free water was used as a negative control.

#### Amplification of *Pfmdr1* and *Pfdhfr* gene fragments

Amplification of the *Pfmdr1* gene was performed in three fragments (FR 1, FR 2 and FR 3). The first fragment contained codons 86 and 184; second fragment contained codons 1034 and 1042; and third fragment contained codon 1246. The reaction primers and conditions for FR1, FR2 and FR3 were as described by Humphrey *et al*. [13]. The following primers and cycling conditions were used. **FR 1**: Primary reaction primers F: AGGTTGAAAAAGAGTTGAAC and R: ATGACACCACAAACATAAAT; reaction conditions were 94°C for 3 minutes/[94°C for 30 seconds, 45°C for 1 minute, 72°C for 1 minute] x 30 cycles/ 72°C for 5 minutes. The nested reaction primers were F: ACAAAAAGAGTACCGCTGAAT and R: AAACGCAAGTAATACATAAAGTC; reaction condition were the same as primary PCR. **FR 2** primers for the primary reaction were F: GCATTTTATAATATGCATACTG and R: GGATTTCATAAAGTCATCAAC; reaction conditions were 94°C for 3 minutes/ [94°C for 30 seconds, 55°C for 1 minute/ 65°C for 40seconds] x 30 cycles/ 65°C for 5minutes/ 15°C for 5 minutes. The nested reaction primers were F: GGTTTAGAAGATTATTTCTGTAA and R: GGATTTCATAAAGTCATCAAC; reaction conditions were the same as the primary reaction. **FR3** primary reaction primers were F: CAAACCAATCTGGATCTGCAGAAG and R: CAATGTTGCATCTTCTCTTCC; the reaction conditions were 94°C for 3 minutes/ [94°C for 30 seconds, 56°C for 1 minute, 65°C for 50 seconds] x 30cycles/ 65°C for 5minutes/ 15^°C^ for 5 minutes. The nested reaction primers were F: GATCTGCAGAAGATTATACTG and R: CAATGTTGCATCTTCTCTTCC; the reaction conditions were the same as primary reaction.

The primers used for sequencing of the three fragments were: FR 1 (F: ACAAAAAGAGTACCGCTGAAT and R: AAACGCAAGTAATACATAAAGTC), FR 2 (F: GGTTTAGAAGATTATTTCTGTAA and R: GGATTTCATAAAGTCATCAAC) and FR 3 (F: GATCTGCAGAAGATTATACTG and R: CAATGTTGCATCTTCTCTTCC). The sequencing reaction conditions were 96°C 1 minute [96°C 30 seconds-50°C-60°C 4 minutes] × 26 cycles.

Amplification of *Pfdhfr* gene involved primers and cycling conditions described by other workers [14,15]. In brief, parasite DNA extracts from blood spots were amplified in a nested polymerase chain reaction. Primary reaction (650 bp) primers were F_*Pfdhfr*_M1 TTT ATG ATG GAA CAA GTC TGC and R_*Pfdhfr*_M7 CTA GTA TAT ACA TCG CTA ACA. The reaction conditions were 93°C for 5 5 minutes, [94°C for 30seconds, 54°C for 60seconds, 65°C for 60seconds] x 41cycles, 65°C for 5 minutes, 15°C for 5 minutes. Nested reaction (594 bp) primers were F_*Pfdhfr*_M3 TGA TGG AAC AAG TCT GCG ACG TT and R_*Pfdhfr*_M9 CTG GAA AAA ATA CAT CAC ATT CAT ATG. The reaction conditions were 95°C for 5 minutes, [93°C for 30 seconds, 56°C for 30 seconds, 68°C for 75 seconds] x 30 cycles, 75°C for 5 minutes, 4°C hold.

All amplicons of the *Pfmdr1* and *Pfdhfr*genes were re-amplified in a nested PCR step. The PCR products of nested reactions were separated by gel electrophoresis on a 1.2% agarose gel stained with ethidium bromide to identify amplified bands of DNA under ultra-violet illumination. Amplicons from nested PCR reactions were purified using the QIAquick PCR Purification Kit (QIAGEN, UK) according to manufacturer’s instructions and subjected to di-deoxy fluorescent sequencing (BigDye 3.1, Applied Biosystems, UK) using conditions and sequencing primer pairs described elsewhere (Pearce *et al*., 2003). The sequence of amplified DNA products was determined using ABI PRISM 3730 Genetic Analyser (Applied Biosystems, UK). ChromasLite® (Technelysium, Australia) was used to analyse the sequence results. The DNA base sequence was converted to peptide sequences using a web-based six frame nucleotide to peptide sequence conversion tool, Transeq® (http://www.ebi.ac.uk/Tools/emboss/transeq/index.html). Detection of single nucleotide polymorphism (SNP) was carried out by aligning the sample protein sequences with reference protein sequence of the *Pfmdr1* and *Pfdhfr* of standard 3D7 (wild) strain of *P*. *falciparum* using the online multiple alignment tool, ClustalW (http://www.ebi.ac.uk/Tools/clustalw2/index.html).

### Data analysis

The data generated from the study were analyzed using EPIINFO^TM^ Version 3.5.1 statistical software (Center for Disease Control, USA). Frequency tables were generated for resistance markers and haplotypes of genes studied. Comparison of key mutations in the genes versus age, gravidity and gestational age of the women was tested by analysis of variance. Associations between parasitaemia and key mutations were tested for significance by the Kruskal Wallis test.

## Results

**Fig 1:**
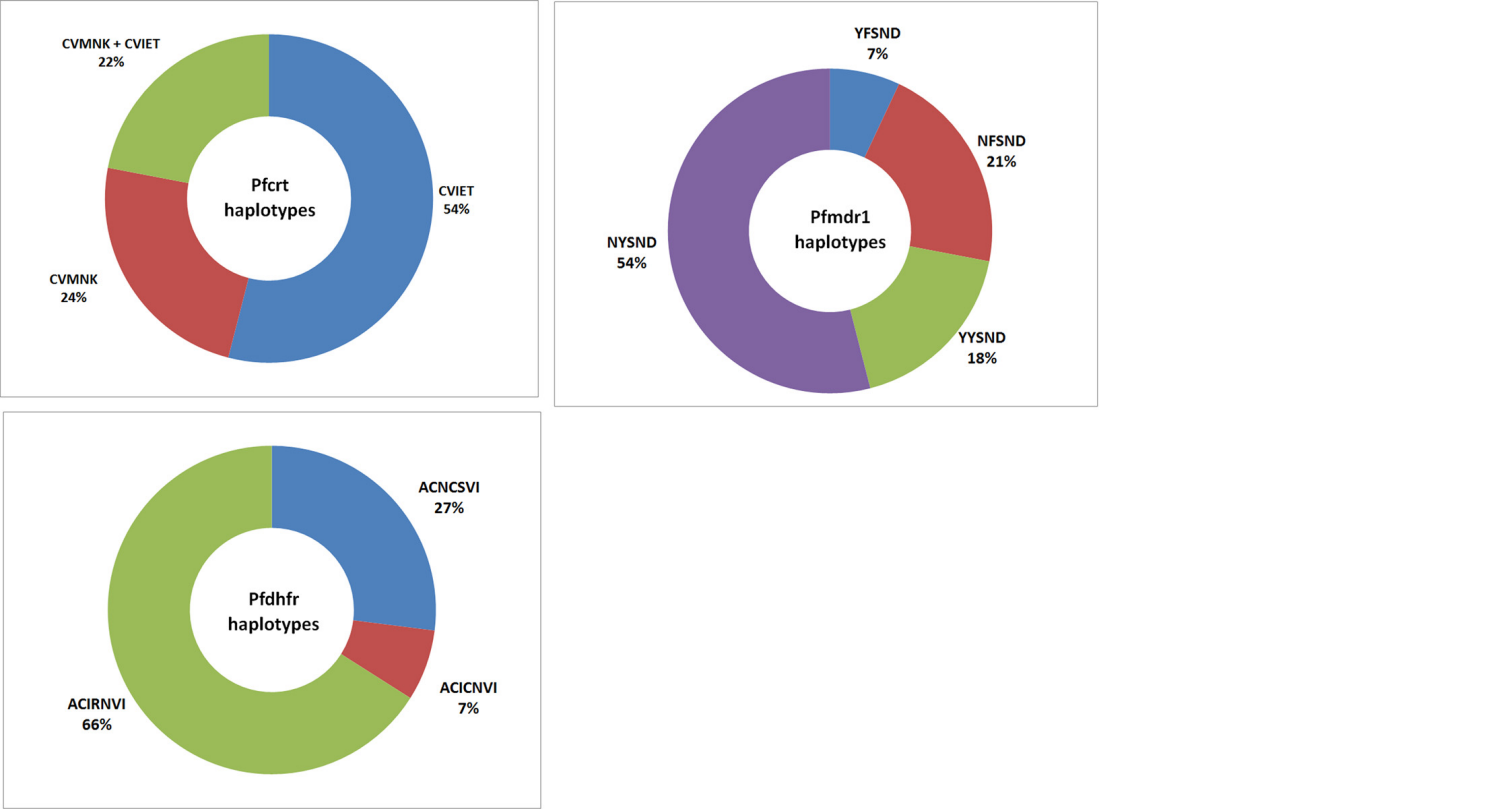
Haplotypes of *Pfcrt*, *Pfmdr1* and *Pfdhfr* genes of *P*. *falciparum* isolates from pregnant women. ***Pfcrt*** (n = 54): *CVMNK* (wild), *CVIET* (mutant); ***Pfmdr1*** (n = 28): NYSND (wild), **Y**YSND (single mutant), N**F**SND (single mutant), **YF**SND (double mutant); ***Pfdhfr*** (n = 15): ACNCSVI (wild), AC**I**C**N**VI (double mutant), AC**IRN**VI (triple mutant).

The haplotype of *Pfcrt* gene was defined by the predicted protein sequence in codons 72–76: CVMNK haplotype is the wild strain while CVIET and SVMNT haplotypes are mutant strains. Thirteen (24.1%) of the 54 evaluable samples had CVMNK strains only, 29(53.7%) had CV**IET** strains only, while 12(22.2%) were mixed with CVMNK and CVIET. Thus, the mutant strains of *P*. *falciparum* were present in 41 (75.9%) of the samples. The **S**VMN**T** haplotype of *Pfcrt* was not found in any of the samples (Fig 1).

The *Pfmdr1* haplotype was defined by the protein sequence at codons 86, 184, 1034, 1042 and 1246 for 28 individuals. The NYSND haplotype (wild) had the highest frequency 15(53.6%). Two types of single mutant haplotypes were observed, YYSND 5(17.9%) and NFSND 6(21.4%). The double mutant haplotype YFSND had the least frequency 2(7.1%) (Fig 1).

The *Pfdhfr* gene haplotype was defined by the protein sequence at codons 16, 50, 51, 59, 108 and 164 for 15 individuals. ACIRNVI (triple mutant) had the highest frequency, 10 (66.7%). The frequency of ACNCSVI (wild) and AC**I**C**N**SVI (double mutant) were 4(26.7%) and 1(6.7%) respectively Mutations were not observed at codons 16, 50 and 164 in this study. (Fig 1).

The rate of occurrence of key single nucleotide polymorphisms in *Pfcrt* (76T) and *Pfdhfr* (108N) were 75.9% and 73.3% respectively. Single nucleotide polymorphisms at codons 86 (86Y) and 184 (184F) of *Pfmdr1*geneoccurred at the rate of 25% and 28.1% respectively.

There was no statistically significant relationship between median parasitaemia (parasites/μl) and genotype for *Pfcrt K76T*, *Pfmdr1 Y184F*, and *Pfdhfr S108N*, but *Pfmdr1 N86Y* was associated with a lower parasitaemia than in infections harbouring wild-type N86 (Table 1). The mutant haplotypes of *Pfcrt K76T*, *Pfmdr1 Y184F and Pfmdr1 N86Y* occurred more in pregnant women with lower mean gestational age but that was not the case for *Pfdhfr S108N*. Furthermore, mutations in *Pfcrt K76T* and *Pfmdr1N86Y* were more common in women with higher gravidity. However, there was no association between gravidity or gestational age of the women and presence of mutations in the *Pfcrt*, *Pfmdr1* or *Pfdhfr* genes (P>0.05) (Tables 2 and 3).

**Table 1:** Relationship between parasitaemia (parasites/μl) and mutations in *Pfcrt and Pfmdr1* and *Pfdhfr* genes.

Gene	Strain	n	Median Parasitaemia	Interquartile range	Kruskal-Wallis	P
*Pfcrt K76T*	Wild (76K)	13	127	81–527	0.904	0.342
* *	Mutant (76T)	41	444	100–1664		
* *	Total	54	364	88–1680		
*Pfmdr1 N86Y*	Wild (86N)	21	476	176–2814	5.084	0.024
* *	Mutant (86Y)	7	71	40–475		
* *	Total	28	400	77–2442		
*Pfmdr1 Y184F*	Wild (184Y)	20	352.5	74–1922	0.372	0.542
* *	Mutant (184F)	8	947.5	128–3197		
* *	Total	28	400	77–2442		
*Pfdhfr S108N*	Wild (108S)	4	294.5	45–1096	0.273	0.602
* *	Mutant (108N)	11	149	84–1419		
* *	Total	15	149	76–1419		

**Table 2:** Relationship between gestational age (months) and mutations in *Pfcrt and Pfmdr1* and *Pfdhfr* genes.

Gene	Strain	n	Mean gestational age	SD	F	P
*Pfcrt K76T*	Wild (76K)	13	5.2	1.8	1.205	0.277
* *	Mutant (76T)	41	4.6	1.6		
* *	Total	54	4.7	1.6		
*Pfmdr1 N86Y*	Wild (86N)	21	5.3	1.8	1.852	0.185
* *	Mutant (86Y)	7	4.3	1.1		
* *	Total	28	5	1.7		
*Pfmdr1 Y184F*	Wild (184Y)	20	5.2	1.6	0.637	0.432
* *	Mutant (184F)	8	4.6	2		
* *	Total	28	5	1.7		
*Pfdhfr S108N*	Wild (108S)	4	3.8	2.4	3.047	0.104
* *	Mutant (108N)	11	5.7	1.8		
* *	Total	15	5.2	2.1		

**Table 3:** Relationship between gravidity and mutations in *Pfcrt*, *Pfmdr1* and *Pfdhfr* genes.

Gene	Strain	n	Mean gravidity	SD	F	P
*Pfcrt K76T*	Wild (76K)	13	1.6	1.2	1.286	0.262
* *	Mutant (76T)	41	2.1	1.2		
* *	Total	54	1.9	1.2		
*Pfmdr1 N86Y*	Wild (86N)	21	2	1.3	0.525	0.475
* *	Mutant (86Y)	7	2.4	1.5		
* *	Total	28	2.1	1.3		
*Pfmdr1 Y184F*	Wild (184Y)	20	2.2	1.3	0.069	0.795
* *	Mutant (184F)	8	2	1.5		
* *	Total	28	2.1	1.3		
*Pfdhfr S108N*	Wild (108S)	4	2.8	1.3	1.682	0.217
* *	Mutant (108N)	11	2	0.9		
* *	Total	15	2.2	1		

Pfcrt K76T mutant occurred more in younger pregnant women compared to the wild-type (P = 0.006). However, the occurrence of the mutant and wild forms of *Pfmdr1N86Y*, *Pfmdr184F* and *Pfdhfr S108N* was similar irrespective of the age of the pregnant women (Table 4).

**Table 4:** Relationship between age (years) and mutations in *Pfcrt and Pfmdr1* and *Pfdhfr* genes.

Gene	Strain	n	Mean Age	SD	F	P
*Pfcrt K76T*	Wild (76K)	13	28.1	5	8.196	0.006
* *	Mutant (76T)	41	24.1	4.5		
* *	Total	54	25.1	4.9		
*Pfmdr1 N86Y*	Wild (86N)	21	23.7	3.8	3.077	0.091
* *	Mutant (86Y)	7	26.9	5.3		
* *	Total	28	24.5	4.3		
*Pfmdr1 Y184F*	Wild (184Y)	20	25.4	4.4	3.592	0.069
* *	Mutant (184F)	8	22.1	3.2		
* *	Total	28	24.5	4.3		
*Pfdhfr S108N*	Wild (108S)	4	24.3	4.8	0.062	0.807
* *	Mutant (108N)	11	24.9	4.4		
* *	Total	15	24.7	4.4		

## Discussion

This is the first study to report on antimalarial drug resistance markers in *P*. *falciparum* isolates from pregnant women in Nigeria. The high rate of mutant haplotypes of *Pfcrt* is an indication of sustained CQ pressure in Lagos. This suspicion is supported by the observation in the phase one results of this study that 20% of the pregnant women had taken chloroquine prophylaxis before booking at the various antenatal clinics [16]. High frequency (82.3%) of the mutant form of *Pfcrt* gene has also been observed in pregnant women taking chloroquine prophylaxis in Senegal [17]. High frequencies of mutant forms of *Pfcrt*, similar to findings of this study among pregnant women, have been reported by other studies in pre-treatment *P*. *falciparum* isolates from children in Southwest Nigeria: 74.0% in Oshogbo, Osun State[18] and 78% in Ibadan, Oyo state [4].

The findings of this study suggests that the use of CQ in this area of Nigeria for either chemoprophylaxis or treatment of malaria during pregnancy may not be effective and should be actively discouraged because it maintains the observed strong selection at the *Pfcrt* locus [17,19]. In Swaziland, *CVIET* haplotype increased in frequency from 70% in 1999 to 83% in 2007 in the presence of sustained chloroquine use [20]. The withdrawal of CQ from the open market should be considered to restrict access to CQ. Re-emergence of CQ-sensitive parasites with CVMNK haplotype of *Pfcrt* gene has been reported following withdrawal of CQ some malaria-endemic countries: Malawi [21,22], Gabon [23], Brazil [24] and China [25]. The absence of SVMNT haplotype observed in this study is consistent with findings of other workers that CVIET is the central determinant of chloroquine resistance in West Africa [26].

Amodiaquine has been reported to select the mutant *Pfmdr1 86Y* in The Gambia [27], Kenya [28] and Nigeria [4]. Thus, the low level of *Pfmdr1 Y86* in this study (25.0%) suggests that the parasites would still be sensitive to amodiaquine for some time. Therapeutic use of drugs such as artesunate-amodiaquine combination in pregnancy is expected to still be effective in Lagos. Moreover, Lekana-Douki et al., [29] reported an association between increase in *Pfmdr1 86N* and introduction of artemisinin-based combination therapies. In Nigeria, ACTs have been in use since 2005 though not on a large-scale at inception. Molecular surveillance is therefore recommended to monitor the *Pfmdr1* haplotypes.

Selection for mefloquine resistance has been associated with a decreased resistance to CQ and an amplification of the *Pfmdr1* gene copy numbers. In areas subjected to mefloquine pressure, wild-type *Pfmdr1* 86N was found more frequently [30]. Although the *Pfmdr1*86N was present in 75% of the isolates in this study, a concrete statement on the resistance cannot be made because the *Pfmdr1* gene copy numbers was not determined.

The lack of association between gravidity and mutations in *Pfcrt*, *Pfmdr1* and *Pfdhfr* codons studied shows that the gravidity of the women did not exert a significant selective pressure. This is consistent with the report that the immune system of semi-immune individuals is able to clear both drug-resistant and sensitive parasites [31].

The *Pfmdr1* 86Y occurred more at very low parasitaemia. This phenomenon was however not observed with *Pfmdr1 and pfdhfr*. This report is at variance with the report of Tukwasibwe et al.[32] that in children mutant type malaria parasites were associated with lower virulence, occurring more in asymptomatic children. The immunological differences between children and pregnant women might explain the difference in the findings. However, a major difficulty for our study was the frequent failure to obtain high quality PCR products from DNA sample from our asymptomatic patients, all of whom has low peripheral parasitaemia. This lead to a great reduction in our sample size for some analyses, and future studies should focus on obtaining larger sample sets, to generate more accurate estimates of marker prevalence.

The triple mutant ACIRNVI haplotype of the *Pfdhfr* gene, which has been reported to be associated with pyrimethamine resistance [33,34], had a high frequency in this population studied. Thus, antimalarial chemoprophylaxis with pyrimethamine in Lagos is not advised. Key mutations such as 16V + 108T and 164L [6,35] associated with high resistance to cycloguanil, the active form of proguanil, were not observed in this study.

## Conclusions

High levels of CQ-resistant haplotypes of *Pfcrt* gene and pyrimethamine-resistant haplotypes of *Pfdhfr* gene were observed in *P*. *falciparum* isolates from pregnant women in Lagos. However, cycloguanil-resistance conferring mutations were not observed in the *Pfdhfr* gene. The low level of mutations in the *Pfmdr1*gene suggests the likely efficacy of amodiaquine against malaria in pregnancy, but future, larger studies are required to confirm this.
